# Ovarian Torsion in a Young Adolescent with Rokitansky Syndrome

**DOI:** 10.1155/2024/1305476

**Published:** 2024-02-22

**Authors:** Francesco Fedele, Giovanna Esposito, Andrea Busnelli, Fabio Parazzini

**Affiliations:** ^1^University of Milan, Milan, Italy; ^2^IRCCS Humanitas Research Hospital, Rozzano, Milan, Italy

## Abstract

A case report of a premenarcheal patient with ovarian torsion and mullerian agenesis is presented. A 12-year-old prepubertal girl is presented with severe right lower quadrant abdominal pain and mild rebound. Laparoscopy showed mullerian agenesis and twisted right adnexa. Detorsion and cystectomy of the right ovary were done, and the ovary was fixed to the pelvic sidewall. The postoperative course was uneventful. An association between the lax attachment of the adnexa and torsion may be a contributing factor in this condition.

## 1. Introduction

The Mayer-Rokitansky-Küster-Hauser (MRKH) syndrome is characterized by congenital absence of the uterus, cervix, and the upper part of the vagina in otherwise phenotypically normal 46,XX females. Its incidence is about 1 in 4,500-5,000 newborn females and is the second most common cause of primary amenorrhea.

Generally, MRKH is divided into two subtypes: MRKH type 1, in which only the upper vagina, cervix, and the uterus are affected, and MRKH type 2, which is associated with additional malformations generally affecting the renal and skeletal systems [1]. Since patients with MRKH syndrome have fully developed secondary sexual characteristics, the main symptom that allows its diagnosis in adolescence is primary amenorrhea. The structure and function of the ovaries are also usually normal, although gonadal dysgenesis and ovarian agenesis have been described in a few cases [2] and sometimes have an extrapelvic location. In all cases, the ovaries are highly mobile, lacking the uteroovarian ligament and having the infundibulopelvic ligament as the only anatomical link, which carries the vasculature and nerves.

We report a rare case of ovarian torsion in a young adolescent. Our case highlights the diagnostic challenge posed in a prepubertal age in association with mullerian agenesis and the benefits of early treatment.

## 2. Case Report

**Figure 1:**
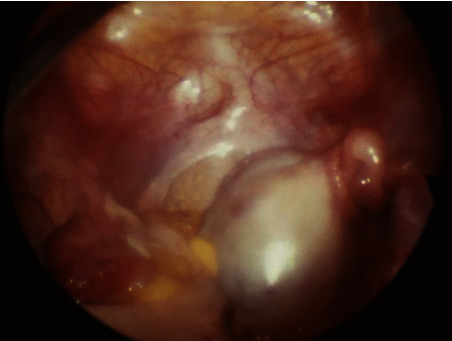
Laparoscopic findings. The uterus is absent. The right ovary, the site of a cystic formation, is twisted on itself together with the tube.

A 12-year-old girl was taken to her trusted pediatrician, complaining of lower abdominal and right iliac fossa pain associated with vomiting. No fever, chill, urinary symptoms, or diarrhea was reported. Her past medical history was unremarkable, and she did not have any history of menstruation, had undergone thelarche at age 10, and had adrenarche at age 11. Blood count, ESR, and CRP were normal, and a diagnosis of mild appendicitis was made. In the following days, the pain gradually increased in severity and the young patient underwent abdominal ultrasound which revealed the presence of a cyst with mixed echogenicity with a diameter of 6 × 4 cm, located in the midline of the pelvis with minimal free fluid. Collaterally, the sonographer reports that he is unable to highlight the uterus, while the organs of the upper abdomen are normal. The torsion of an ovary containing a cystic formation is therefore suspected. A surgical consultation was requested, and the patient is admitted to our institution. On physical examination, appearance and anxiety evidenced that the adolescent was in obvious pain. Abdominal examination revealed a treatable abdomen, with rebound pain and normal bowel sounds. Her external genitalia were normal. A pelvic examination with a cotton swab revealed a blind terminal vagina, 1 cm deep. Rectal examination revealed the presence in the pelvis of a tense elastic mass, painful on mobilization, about 6 cm in diameter, without any blood in the stool. Given the clinical picture, a prompt surgical exploration was decided rather than repeat subjecting the patient to an MRI or other imaging. Laparoscopy confirmed the right ovarian torsion with the tube. Her ovary was rotated 180° (2 full turns). Ovarian tissue still appears viable and has a normal appearance. The uterus was absent, and in its place were two small-volume rudimentary horns, apparently not cavitated, joined by a horizontal fibrous band that separated the bladder from the Douglas cavity (Figure 1). The right ovary and tube were successfully untwisted. The functional cyst contained in the ovary was enucleated, and the ovarian parenchyma was gently coagulated and finally fixed to the right pelvic sidewall. There were no surgical complications, and the patient recovered quickly and fully after surgery. The result of pathology reported a simple cyst of the right ovary. During follow-up, the examinations revealed a normal anatomy of the spines and a 46,XX karyotype of the patient.

## 3. Discussion

The particularities of our case consist in the coexistence of ovarian torsion and a mullerian agenesis, in the timely recourse to laparoscopy without waiting for sophisticated imaging investigations and in the successful conservative treatment which avoided ovariectomy in a young adolescent already marked by the absence of the uterus and the vagina. A few studies have described an association between mullerian duct agenesis and an increased risk of ovarian torsion (Table 1). It is believed that the combination of mullerian uterine agenesis and the absence of the uteroovarian ligament results in increased ovarian mobility [3–6]. Under normal conditions, the ovaries are suspended in place by infundibulopelvic ligament, uteroovarian ligament, and mesosalpinx. Without a uterus, in the case of a patient with mullerian agenesis, the ovary is only suspended in place by the infundibulopelvic ligament attaching it to the pelvic wall since the fallopian tubes may provide little fixation. As a result of nonfixation, the ovaries may be more prone to undergo torsion. Adnexal torsion is the fifth most common gynecologic emergency with a reported incidence of 3% in one series of acute gynecologic complaints. Torsion is usually facilitated by an anatomic change in the size such as a tubal or ovarian cyst, although some cases have been reported in patients with normal adnexa. The benign ovarian expansive pathology is most likely to occur at or before the age of reproduction. In our case too, ovarian activity had begun, only that there had been no menstrual flows due to uterine agenesis. Ovarian torsion is a gynecologic emergency in young women and should be considered in a patient with a sudden onset of pelvic pain. Moreover, all forms of torsion should be suspected in adolescents with primary amenorrhea and acute abdominal pain, since it has been already reported a case of hematosalpinx torsion in patients with MRKH syndrome and active uterine remnants [7]. Preoperative diagnosis of adnexal torsion is often difficult. Therefore, it is imperative to maintain a high index of suspicion when evaluating pediatric and adolescent patients. In fact, signs of the physical examination are frequently indistinguishable from other conditions, such as acute appendicitis, when the right side is involved. If torsion cannot be excluded, surgical intervention is recommended for diagnosis. We managed our case conservatively with untwisting of the ovary and fixing it to the left abdominal wall. This approach has previously been reported in multiple series with adequate preservation of ovarian function. Detorsion of the gonad ensures an adequate restoration of venous blood supply, lymphatic drainage, and arterial supply. When treating premenarcheal patients, this approach should be considered to prevent future loss of ovarian function. In our patient, although a mullerian anomaly would prevent her from carrying a pregnancy, the possibility of assisted reproductive technique may allow her to have offsprings carried by a surrogate mother.

## 4. Conclusions

We have presented the case of a woman with MRKH syndrome with an ovarian torsion. This association can be explained by the increased mobility of the ovaries in mullerian agenesis. Clinicians should be alert to this association so that they can intervene early if ovarian torsion is suspected.

## Figures and Tables

**Table 1:** Rokitansky syndrome and ovarian torsion.

Author, year	Patient's age (years)	Torsion (adnexal/ovarian)	Surgery (laparoscopy/laparotomy)	Treatment (conservative/radical)
Masoumi Shahrbabak and Ebrahimi Meimand, 2021 [3]	9 y	Right ovary in inguinal hernia (ovary with simple cyst)	Laparotomy	Radical
Morabito et al., 2020 [4]	4 y	Right ovary in inguinal hernia	Laparotomy	Radical
Sullivan et al., 2012 [5]	14 y	Right tube and ovary (ovary with 2 large cysts)	Laparotomy	Radical
Lara-Torre et al. 2005 [6]	11 y	Left adnexa (ovary with multiple cysts)	Laparoscopy	Conservative
